# Molecular epidemiology and phylogenomic analysis of *Mycobacterium abscessus* clinical isolates in an Asian population

**DOI:** 10.1099/mgen.0.000708

**Published:** 2021-11-30

**Authors:** Ka Lip Chew, Sophie Octavia, Roland Jureen, Oon Tek Ng, Kalisvar Marimuthu, Raymond Tzer Pin Lin, Jeanette W. P. Teo

**Affiliations:** ^1^​ Department of Laboratory Medicine, National University Hospital, Singapore; ^2^​ Environmental Health Institute, National Environment Agency, Singapore; ^3^​ National Centre for Infectious Diseases, Singapore; ^4^​ Department of Infectious Diseases, Tan Tock Seng Hospital, Singapore; ^5^​ Yong Loo Lin School of Medicine, National University of Singapore, Singapore; ^6^​ National Public Health Laboratory, National Centre for Infectious Diseases, Singapore

**Keywords:** genomic epidemiology, *Mycobacterium abscessus *complex, *Mycobacterium bolletii *, *Mycobacterium massiliense*, whole genome sequencing

## Abstract

*
Mycobacterium abscessus* comprises three subspecies*: M. abscessus subsp. abscessus* *, M. abscessus subsp. bolletii* *, and M. abscessus subsp. *massiliense **. These closely related strains are typically multi-drug-resistant and can cause difficult-to-treat infections. Dominant clusters of isolates with increased pathogenic potential have been demonstrated in pulmonary infections in the global cystic fibrosis (CF) population. An investigation was performed on isolates cultured from an Asian, predominantly non-CF population to explore the phylogenomic relationships within our population and compare it to global *M. abscessus* isolates. Whole-genome-sequencing was performed on *M. abscessus* isolates between 2017 and 2019. Bioinformatic analysis was performed to determine multi-locus-sequence-type, to establish the phylogenetic relationships between isolates, and to identify virulence and resistance determinants in these isolates. A total of 210 isolates were included, of which 68.5 % (144/210) were respiratory samples. These isolates consisted of 140 (66.6 %) *M *. *abscessus* subsp. *massiliense *, 67 (31.9 %) *M *. *abscessus* subsp. abscessus*,* and three (1.4 %) *M *. *abscessus* subsp. *bolletii *. Dominant sequence-types in our population were similar to those of global CF isolates, but SNP differences in our population were comparatively wider despite the isolates being from the same geographical region. ESX (ESAT-6 secretory) cluster three appeared to occur most commonly in ST4 and ST6 *M. abscessus* subsp. *massiliense *, but other virulence factors did not demonstrate an association with isolate subspecies or sample source. We demonstrate that although similar predominant sequence-types are seen in our patient population, cross-transmission is absent. The risk of patient-to-patient transmission appears to be largely limited to the vulnerable CF population, indicating infection from environmental sources remains more common than human-to-human transmission. Resistance and virulence factors are largely consistent across the subspecies with the exception of clarithromycin susceptibility and ESX-3.

## Data Summary

Raw sequence reads and assemblies all *M. abscessus* subspecies in this study have been submitted to GenBank under project accession number PRJNA734660.

**Impact Statement**

*
M. abscessus* cultured from our Asian population were dominated by the same sequence type (ST) profiles seen in global cystic fibrosis (CF) populations. However, unlike in some CF centres cross-transmission with clonal isolates were not demonstrable. The infection control risks appear to be largely limited to the vulnerable CF population, indicating that infection from environmental sources is the most likely route rather than human-to-human transmission in our setting.

## Background

Non-tuberculous-mycobacteria (NTM) are environmental organisms that may result in human infections in vulnerable patient groups. *Mycobacterium abscessus* comprises three subspecies: *M. abscessus* subsp. abscessus*, M. abscessus subsp. bolletii* *,* and *M. abscessus* subsp. *massiliense *. Inoculation of the bacteria following trauma or surgery may result in skin and soft tissue infections [1]. Respiratory infections usually occur in patients with underlying lung disease such as bronchiectasis, chronic obstructive pulmonary disease, and cystic fibrosis [1].

Treatment of infections of *M. abscessus* is fraught with difficulties due to significant antimicrobial resistance. Two guidelines are available for treatment of pulmonary infections with *M. abscessus *: one by the British Thoracic Society, and a joint recommendation made by the American Thoracic Society (ATS), European Respiratory Society (ERS), European Society of Clinical Microbiology and Infectious Diseases (ESCMID), and the Infectious Diseases Society of America (IDSA) [2, 3]. Multidrug treatment regimens are usually recommended, with macrolides (clarithromycin or azithromycin) and amikacin being some of the key drugs used. Clarithromycin susceptibility and administration of clarithromycin has a significant impact on treatment outcomes. The impact of other antibiotics on outcome is less clear. Treatment outcomes remain poor despite long periods of treatment. It should also be noted that current guidelines and clinical data is largely based on data from pulmonary *M. abscessus* infections [2, 3]. Antimicrobials typically recommended for pulmonary infections may have different pharmacokinetic profiles for other infection sites and additional clinical data is required to guide management of extrapulmonary infections.

Unlike tuberculosis, *M. abscessus* (and NTM in general) are environmental organisms, and human-to-human spread is thought to be limited. However, there is increasing evidence of potential human-to-human transmission, with whole-genome-sequencing data used demonstrating closely-related *M. abscesuss* isolates within CF centres [4–6]. In conjunction with epidemiological links, these likely represent transmission between patients. CF is the most common predisposing risk factor for pulmonary *M. abscessus* infections in Caucasian populations [4, 7]. Consequently, genomic analyses of clinical *M. abscessus* isolates have largely centred on isolates from CF patients [4]. Transmission is postulated to have occurred via generation of infectious aerosols by infected patients, and fomites [4]. Bryant *et al.* demonstrated that among global collection of *M. abscessus* isolates from CF patients, the majority of isolates formed three major clusters: two *M. abscessus* subsp. *abscessus* and one *M. abscessus* subsp. *massiliense* clusters [4]. Higher rates of phagocytosis and intracellular survival were demonstrated among clustered isolates when compared against unclustered isolates, suggesting higher pathogenic potential [4]. Murine models also demonstrated higher intracellular bacterial survival, higher bacterial burdens, and worse inflammation when infected with clustered isolates [4].

The pathology and virulence factors of *M. abscessus* is an under-studied field. Emerging evidence that intracellular virulence factors, namely the type VII secretion systems encoded by different ESX (ESAT-6 secretory) clusters, in particular ESX-3 and ESX-4 which appear to be unique to *M. abscessus* subsp. *abscessus* [8], the glycopeptidolipid (*gpl*) locus which encompasses a large set of lipid membrane transport proteins (MmpL-MmpS), and phospholipase C are important for pathogenesis [9].

Compared to Caucasian populations, CF is rare in Asians [10]. The findings of previous studies thus may not be applicable to Asian populations, particularly as comprehensive and systematic genomic analyses of *M. abscessus* from Asian populations are limited. Several questions remain unanswered such as whether there are also clustered clinical *M. abscessus* isolates in Asian populations, and whether there is similar pathogenic potential in these isolates. These could provide clues to further elucidate potential channels of transmission and pathogenicity in different patient populations. Whole-genome-sequencing of a collection of isolates from an Asian patient population was performed to explore the phylogenomic relationships within our population and compared to global *M. abscessus* isolates. Potential resistance mutations were also explored.

## Methods

Clinical *M. abscessus* isolates cultured between 1 January 2017 and 31 December 2019 for which susceptibility testing was performed were included in this study. The isolates were identified previously by Bruker MALDI Biotyper (Bruker, Billerica, Massachusetts, US). Susceptibility testing was performed during this period if microbiological criteria for pulmonary samples were fulfilled (>1 positive respiratory culture from the same patients, sample positive from a bronchoalveolar lavage). All non-pulmonary samples had susceptibility testing performed. Only the first sample for which susceptibility testing was performed was included from each patient.

The phenotypic susceptibility testing results of these isolates have been previously performed and reported [11]. This include routinely tested antimicrobials (RAPMYCO plate, Sensititre, Thermo Fisher, Waltham, Massachusetts US: trimethoprim-sulfamethoxazole ciprofloxacin, moxifloxacin, cefoxitin, amikacin, doxycycline, tigecycline, clarithromycin, linezolid, imipenem, minocycline, and tobramycin), and an extended antimicrobial panel using a customized antibiotic panel (SGPNUHS1 plate, Sensititre : vancomycin, oritavancin, dalbavancin, telavancin, rifabutin, eravacycline, delafloxacin, clofazimine, and bedaquiline). In brief, testing was performed as per manufacturer instructions and incubated at 30 °C (ambient conditions). Plates were read at 3–5 days’ incubation based on whether sufficient growth was seen in the control wells. RAPMYCO plates were further incubated to 14 days if initial reading indicated clarithromycin susceptibility to exclude presence of inducible clarithromycin resistance. Where available, the MIC results were interpreted based on CLSI breakpoints [12].

### Whole-genome sequencing and bioinformatic analysis

Total genomic DNA was extracted from plate cultures using the QIAamp DNA Mini Kit (Hilden, Germany). Sequencing libraries were prepared using the NexteraXT kit (Illumina Inc., San Diego, CA, USA) and sequenced on the Illumina platform (HiSeq). Raw reads were trimmed using Trimmomatic v. 0.38 [13] then assembled with SPAdes version 3.9.0 [14]. Genome annotation was carried out using Prokka [15]. ABRicate using ResFinder database was used for genetic prediction of both acquired and chromosomal antibiotic resistance determinants. Multilocus sequence typing (MLST) was based on the PubMLST (https://pubmlst.org/mabscessus/) scheme using seven genes (*argH*, *cya*, *gnd*, *murC*, *pta*, *purH*, and *rpoB*) and performed using the MLST software available at https://githubcom/tseemann/mlst. Clustered isolates from a global CF *M. abscessus* collection (European Nucleotide Archive under project accession ERP001039) were also obtained and analysed [4]. The isolates used in our analysis are listed on Table S1 (available in the online version of this article). As Bryant *et al.* [4] did not employ the seven gene MLST scheme, we took the assembled genomes described in Table S1 in order to obtain the sequence types.

For species identification, average nucleotide identity (ANI) values were calculated using the Pyani package (https://github.com/widdowquinn/pyani). The following reference genomes were used for ANI comparisons *M. abscessus* subsp. *abscessus* ATCC 19977 (GenBank:GCA_000069185.1), *M. abscessus* subsp. *bolletii* BD (GenBank:GCA_003609715.1) and *M. abscessus* subsp. *massiliense* str. GO 06 (GenBank:GCA_000277775.2).

For the detection of variants in drug-resistance associated genes (Table 1) Snippy v4.3.0 (https://github.com/tseemann/snippy) was used while the presence and absence of putative virulence factors were determined using custom database coupled with ABRicate. FastTree [16] was used to generate phylogenetic tree based on core genome-SNPs obtained from alignment of the draft genomes using Snippy pipeline. The phylogenetic tree was visualized and annotated using iTOL [17].

**Table 1.** List of resistance loci screened in the study genomes

Phenotypic resistance to	Gene	Product	* M. abscessus* subsp. *abscessus* ATCC 19977 locus tag
Inducible macrolide resistance	*erm*(41)	23S rRNA methyltransferase	MAB_2297
Constitutive macrolide resistance	*rrl*	23S ribosomal RNA	MAB_r5052
Aminoglycoside	*rpsL*	30S ribosomal protein S12	MAB_3851c
Aminoglycoside	*rrs*	16S ribosomal RNA	MAB_r5051
Amikacin	*eis1*	Gcn5-related N-acetyltransferase	MAB_4124
Amikacin	*eis2*	Gcn5-related N-acetyltransferase	MAB_4532c
Clofazimine and bedaquiline	MAB_2299c	Transcriptional regulatory protein	MAB_2299c
Clofazimine and bedaquiline	MmpS-MmpL	Membrane protein	MAB_2300 – MAB_2301
Clofazimine and bedaquiline	MmpS-MmpL	Membrane protein	MAB_1135c – MAB_1134c
Tetracycline	*tetX*	FAD-binding monooxygenase	MAB_1496c
Tetracycline	*tetR*	TetR regulatory protein	MAB_1497c
Tigecycline	*whiB7*	Transcriptional regulator	MAB_3508c
Rifampicin	MAB_0591	Rifampin adp-ribosyl transferase	MAB_0591
Multi-drug	MAB_2780c	Transporter	MAB_2780c
Multi-drug	MAB_2958	Putative transmembrane-transport protein	MAB_2958
Multi-drug	MAB_1935	Putative drug resistance transporter	MAB_1935

### Data availability

Raw sequence reads and assemblies all *M. abscessus* in this study have been submitted to GenBank under project accession number PRJNA734660.

## Results

**Fig. 1.**
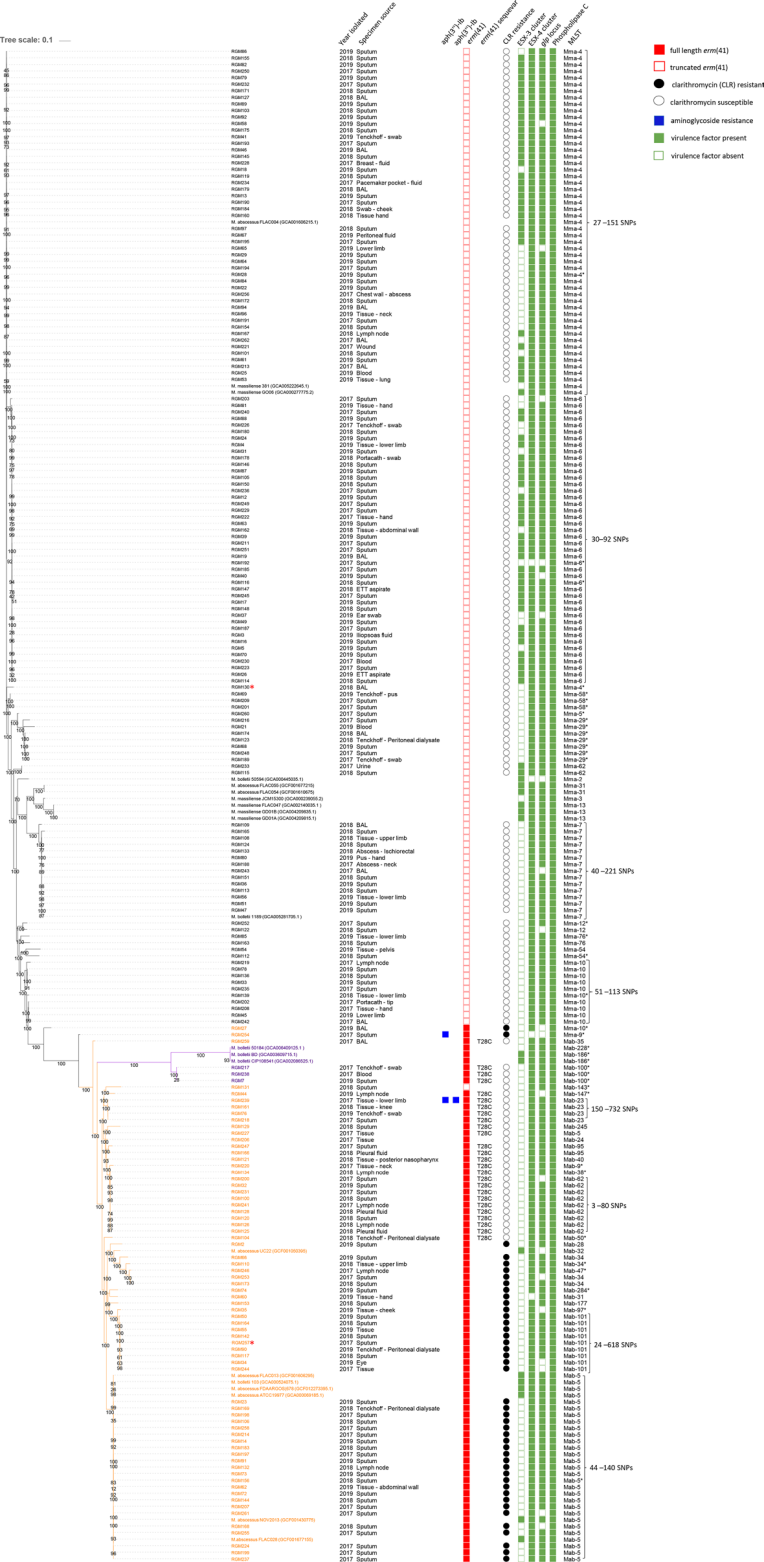
Core SNP phylogenetic tree of 210 isolates of *Mycobacterium abscessus *. The metadata includes specimen source, resistance determinants, virulence factors and multi-locus sequence type (ST). The black branch labels belong to *M. abscessus* subsp. massiliense*,* the purple labels to *M. abscessus* subsp. *bolletii* and the orange labels to *M. abscessus* subsp. massiliense*.* Two isolates marked with red asterisks were from cystic fibrosis (CF) patients*. glp;* glycopeptidolipid, ESX; ESAT-6 secretion system. The bootstrap values are indicated on the nodes.

Between 1 January 2017 and 31 December 2019, a total of 819 *M. abscessus* isolates were cultured from 506 patients. Susceptibility testing was performed on 268 isolates from 218 patients. A total of 210 non-duplicate isolates were included, of which 68.5 % (144/210) were respiratory samples (bronchoalveolar lavage, sputum, lung and tracheal aspirates) (Fig. 1). Only two isolates were from patients with cystic fibrosis.

An average sequencing depth of 150× was achieved for the genomes. Phylogenetic analysis classified 140 (66.6 %) as *M. abscessus* subsp. *massiliense *, 67 (31.9 %) as *M. abscessus* subsp. *abscessus* and three (1.4 %) as *M. abscessus* subsp. *bolletii* (Fig. 1). The species identification was also supported by ANI values which were typically >99 % when compared to their species reference genome (Table 2).

**Table 2.** Average nucleotide identity (ANI) values of *Mycobacterium abscessus* subspecies

	Study isolates			Reference genomes		
	* M. abscessus* subsp. *abscessus* (*n*=69)	* M. abscessus* subsp *. massiliense* (*n*=138)	* M. abscessus* subsp. *bolletii* (*n*=3)	* M. abscessus* subsp. *abscessus* ATCC19977 (ASM6918v1)	* M. abscessus* subsp. *massiliense* GO06 (ASM27777v2)	* M. abscessus* subsp *. bolletii* BD (ASM360971v1)
* M. abscessus* ATCC19977 (ASM6918v1)	99.3–99.9	97.4–97.5	97.4–97.6	100	97.4	97.4
* M. abscessus* subsp. *massiliense* GO06 (ASM27777v2)	97.3–97.5	99.0–99.9	97.1	96.9	100	97.4
* M. abscessus* subsp. *bolletii* BD (ASM360971v1)	98.2	96.9–97.2	98.6	97.4	96.9	100

GenBank assembly accession numbers are provided for the reference genomes

n, number of study isolates.

**Fig. 2.**
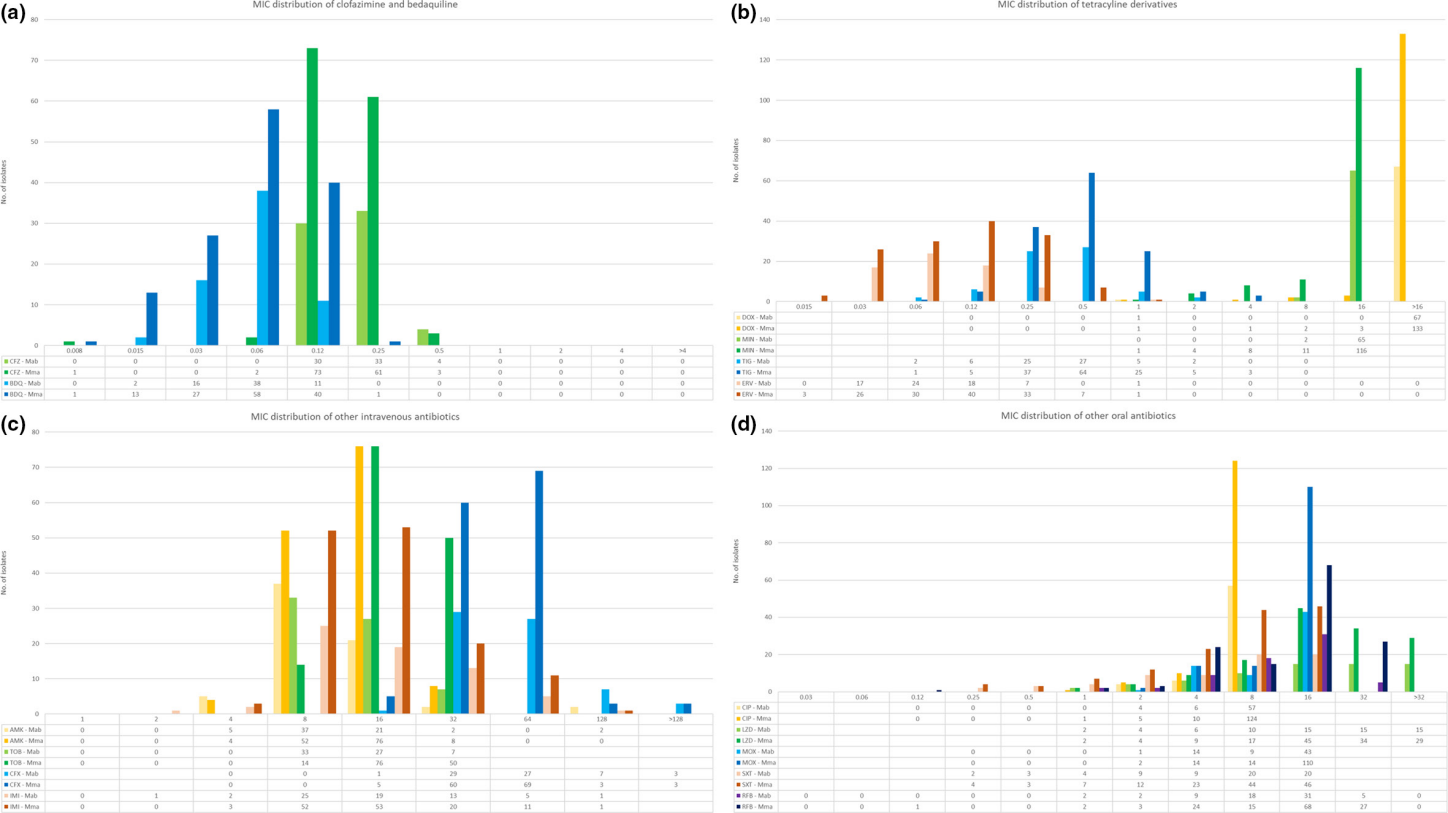
MIC distribution of tested antimicrobials stratified by subspecies. The figures beneath the histograms indicate the number of isolates with a particular MIC, with the right-most figure indicating no inhibition within the tested MIC range. Blank results indicate MICs outside of the tested ranged. MICs presented in mg l^−1^; AMK: Amikacin; CFX: Cefoxitin; CIP: Ciprofloxacin; DOX: Doxycycline; IMI: Imipenem; LZD: Linezolid; MOX: Moxifloxacin; SXT: Co-trimoxazole; TOB: Tobramycin; MIN: Minocycline; TIG: Tigecycline; CFZ: Clofazimine; BDQ: Bedaquiline; ERV: Eravacycline; RFB: Rifabutin; Mab: *Mycobacterium abscessus* subsp. abscessus*;* Mma: *Mycobacterium abscessus* subsp. *massiliense *.

The overall susceptibility testing results have been previously reported without differentiating into the subspecies [11]. With the exception of clarithromycin (Fig. 1), the MIC results did not differ between the two predominant subspecies, *M. abscessus* subsp. *abscessus* and *M. abscessus* subsp. massiliense*.* The susceptibility profiles are summarized in Table 3 with MIC distributions presented in Fig. 2, stratified by subspecies level identification. *M. abscessus* subsp. *bolletii* was not included in this comparison due to lower numbers. Analyses of resistance mechanisms were performed for *M. abscessus* as a whole given the overlapping MIC range of *M. abscessus* subsp. *abscessus* and *M. abscessus* subsp. massiliense*.*

**Table 3.** Minimum inhibitory concentration of *Mycobacterium abscessus* subsp. *abscessus* (*n*=67) and *Mycobacterium abscessus* subsp. *massiliense* (*n*=140)

Antibiotic	Organism	MIC_50_	MIC_90_	Sensitive	Intermediate	Resistant
Amikacin	Mab	16	16	94.0 %	3.0%	3.0%
	Mma	16	32	94.3 %	5.7%	0.0%
Cefoxitin	Mab	64	64	1.5 %	83.6%	14.9%
	Mma	64	64	3.6 %	92.1%	4.3%
Ciprofloxacin	Mab	>4	>4	0.0 %	6.0%	94.0%
	Mma	>4	>4	0.7 %	3.6%	95.7%
Doxycycline	Mab	>16	>16	0.0 %	0.0%	100.0%
	Mma	>16	>16	0.7 %	0.7%	98.6%
Imipenem	Mab	16	32	4.5 %	66.7%	28.8%
	Mma	15	32	2.1 %	75.0%	22.9%
Linezolid	Mab	16	>32	32.8%	22.4%	44.8%
	Mma	16	>32	22.9%	32.1%	45.0%
Moxifloxacin	Mab	>8	>8	0.0%	1.5%	98.5%
	Mma	>8	>8	0.0%	1.4%	98.6%
Trimethoprim-sulphamethoxazole	Mab	8	>8	26.9%	n/a	73.1%
	Mma	8	>8	18.7%	n/a	81.3%
Tobramycin	Mab	16	>16	0.0%	0.0%	100.0%
	Mma	16	>16	0.0%	0.0%	100.0%
Minocycline	Mab	>8	>8	0.0%	0.0%	100.0%
	Mma	>8	>8	0.7%	8.6%	90.7%
Tigecycline	Mab	0.5	1	n/a	n/a	n/a
	Mma	0.5	1	n/a	n/a	n/a
Clofazimine	Mab	0.25	0.25	n/a	n/a	n/a
	Mma	0.12	0.25	n/a	n/a	n/a
Bedaquiline	Mab	0.06	0.12	n/a	n/a	n/a
	Mma	0.06	0.12	n/a	n/a	n/a
Eravacycline	Mab	0.06	0.25	n/a	n/a	n/a
	Mma	0.12	0.25	n/a	n/a	n/a
Rifabutin	Mab	16	16	n/a	n/a	n/a
	Mma	16	32	n/a	n/a	n/a

MIC distribution in mg l^−1^; MIC_50_: MIC required to inhibit the growth of 50 % of included isolates; MIC_90_: MIC required to inhibit the growth of 90 % of included isolates. Mab: *M. abscessus* subsp. abscessus*;* Mma: *M. abscessus* subsp. *massiliense*. N/A, CLSI and EUCAST interpretive breakpoints not available.

n, number of study isolates.

### Multi-locus-sequence-typing (MLST)

For *M. abscessus* subsp. *massiliense *, a total of 18 sequence types (STs) were detected amongst the 140 isolates. This included ten novel STs. On average, the SNP range for each *M. abscessus* subsp. *massiliense* cluster ranged from five to 200 SNPs (Fig. 1). The most common STs were ST4, ST6, ST7 and ST10 (35, 30, 10 and 6.4 %, respectively). ST4 and ST6 belonged to clonal complex four whilst ST7 and ST10 belonged to clonal complex six and two, respectively. ST4, ST6, ST7, and ST10 all formed clusters identified in *M. abscessus* subsp. *massiliense* in the global CF population, with ST4 being the most common (18.8%, 48/256) [4]. *M. abscessus* subsp. *abscessus* (*n*=67) isolates appeared to have greater ST diversity with 24 different STs detected including ten novel STs. ST5, ST101, ST62, ST23 (32.8, 13.4, 13.4 and 5.9 %, respectively) were amongst the most common with none belonging to the same clonal complex. ST5 was the dominant clone (16.3%, 119/730) found in the previous study on global isolates [4].

Overall, we did not observe an association between specific STs and specimen sources. No predilection of dominant clones for pulmonary infections was seen (Fig. 1). There were four blood culture isolates which belonged to different STs (* M. abscessus* subsp. abscessus* n*=1, *M. abscessus* subsp. massiliense* n*=3). There were also ten peritoneal dialysate peritonitis isolates (* M. abscessus* subsp. abscessus* n*=5, *M. abscessus* subsp. massiliense* n*=5) which also had unique STs, indicating they were not clonally related (Fig. 1).

### SNP analysis

Analysis of isolates from same patients by Bryant *et al.* [4] used 20 SNPs as the cut-off for ‘probable’ patient-patient transmission, and 38 SNPs as the cut-off for ‘possible’ recent transmission. Using these criteria, some of the isolates in our population may meet criteria for *probable/possible* transmission. These include *M. abscessus* subsp. *massiliense* ST4 (8–151 SNPs), ST6 (12–92 SNPs), and *Mycobacterium abscessus* ST62 (3–80 SNPs), ST101 (24–618 SNPs). Although some appear to be closely related, acquisition from the same source due to geographical proximity cannot be excluded. *M. abscessus* subsp. *massiliense* ST7 (40–221 SNPs) ST10 (51–113 SNPs), *M. abscessus* subsp *. abscessus* ST23 (150–732 SNPs), and ST5 (44–140 SNPs) did not meet the criteria for possible recent transmission.

All isolates with <20 SNPs were reviewed for possible links, including four ST62 *M. abscessus* subsp. abscessus*,* and twelve *M. abscessus* subsp. *massiliense* (seven ST6, three ST7, two ST4). The two ST4 *M. abscessus* subsp. *massiliense* were cultured from samples received from two separate external hospitals, 22 months apart. The three ST7 *M. abscessus* subsp. *massiliense* were cultured from internally received samples (*n*=2), and one external sample, received over 2 years. The seven ST6 *M. abscessus* subsp. *massiliense* were cultured from internally received samples (*n*=4), and another external hospital (*n*=3). These were received over 2 years, with the shortest interval being 15 days apart. The four ST62 *M. abscessus* subsp. *abscessus* were cultured from one internally received sample, and two other external hospitals (*n*=3). These samples were received within twelve days. As the isolates were received from different hospitals epidemiological links between these isolates were unlikely and the close relationship (SNPs <20) may not represent transmission events whether between individuals. Transmission from a single point-source may be possible but cannot be confirmed.

### Distribution of virulence factors

Virulence genes ESX-3 and ESX-4, the *glp* locus and phospholipase C were sought in the genomes of our isolates. Complete modules of the ESX-3 system were not detected in *M. abscessus* subsp. *abscessus* genomes as determined by blast of all the loci (data not shown), and present in only 68 *M. abscessus* subsp. massiliense*.* Of note, ESX-3 was most commonly identified in ST4 and ST6 *M. abscessus* subsp *. massiliense* isolates. ESX-4 and *gpl* were not identified in three and eight isolates, respectively, while phospholipase C was ubiquitous in all isolates (Fig. 1). We did not observe a correlation between the distribution of virulence genes and subspecies or isolates from particular specimen sites (Fig. 1).

### Resistance determinants in *M. abscessus*

Clarithromycin resistance in the *M. abscessus* subsp. *abscessus* can be constitutive or inducible. Constitutive clarithromycin resistance attributed to *rrl* mutations (typically point mutations at positions 2058 and 2059 [18]) were not observed in any isolate. All *M. abscessus* subsp. *massiliense* isolates had truncated *erm*(41) gene concordant with their susceptible phenotypes (MICs 0.06–1 mg l^−1^), which is characteristic of this subspecies [19]. All *M. abscessus* subsp. *abscessus* isolates carried the full-length *erm*(41) gene of which 26 (38.8 %, 26/67) isolates were of the C28 sequevar resulting in a non-functional *erm*(41) producing a clarithromycin-sensitive phenotype (Fig. 1). Other previously described sequevars were not observed [20]. All three *M. abscessus* subsp. *bolletii* isolates carried the full-length *erm*(41) with C28 sequevar, with phenotypic susceptibility to clarithromycin.

Resistance to aminoglycosides is conferred by several mechanisms, including target mutation, drug modification, and reduced uptake and/or increased efflux [9]. Aminoglycosides-modifying enzymes are found in *M. abscessus* these include acetyltransferases - AAC(2′), phosphotransferase - APH(3″) and N‐acetyltransferase Eis2 [9]. Out of the 210 genomes, phosphotransferases were detected in only two *M. abscessus* subsp. *abscessus* isolates. These were aph(3'')-Ic and aph (6)--Id, in RGM254 and RGM239, respectively. Both had amikacin MICs of 8 mg l^−1^ and the phosphotransferases did not appear to confer significant aminoglycoside resistance. Target site mutations of *rrs* and *rpsL* are responsible for high-level amikacin resistance in *M. abscessus* subsp. abscessus*.* Two isolates RGM25 and RGM172 had amikacin MICs of 128 mg l^−1^ however no mutations observed in *rrs* and *rpsL* indicating the possibility of other resistance mechanisms.

Overexpression of the *eis2* and the multidrug efflux transporter gene (*tap*) and transcriptional regulator gene *whiB7* have been demonstrated to be involved in the amikacin resistance in *M. abscessus* subsp. *abscessus* [21] although overexpression analysis was not investigated in this study.

Loci contributing to resistance in clofazimine, bedaquiline, tetracycline, and rifamycins (Table 1) were examined [22]. MAB_2299c which encode a putative TetR transcriptional regulator controls the expression of two separate two separate MmpS – MmpL efflux pumps (MAB_2300 – MAB_2301 and MAB_1135c-MAB_1134 c) [23, 24] (Table 1). Point mutations or deletion in MAB_2299 c were commonly associated with clofazimine resistance as well as cross-resistance to bedaquiline. High levels of tetracycline and doxycycline resistance typically seen in *M. abscessus* are conferred by a monooxygenase, TetX (MAB_1496 c), whose expression is induced by the same antibiotics [25]. ADP-ribosyltransferase MAB_0591 is recognized as the major determinant of innate high-level rifamycin resistance in *M. abscessus* [26]. Overall, no meaningful SNPs (Table S2) were detected in these loci and this was reflected in the antibiograms.

## Discussion

The proportion of subspecies observed here mirrored our previous study where *M. abscessus* subsp. *massiliense* was the dominant subspecies among *M. abscessus* identified in our laboratory [27]. Even though the prevalence of each subspecies varies geographically, in most institutions *M. abscessus* subsp. *abscessus* is usually predominant and accounted for 51–78 % of the *M. abscessus *, followed by *M. abscessus* subsp. *massiliense* and *M. abscessus* subsp. *bolletii* [28, 29]. *M. abscessus* subsp. *massiliense* is associated with higher treatment success rates which has been attributed to clarithromycin susceptibility. In addition, the C28 sequevar was seen in a significant proportion (38.8 %) of our *M. abscessus* subsp. *abscessus *. A number of Asian studies have now shown that the C28 sequevar form a sizeable portion of isolates carrying *erm*(41)*.* Studies from South Korean and Taiwan demonstrate that 20 and 37.5 % of their respective *M. abscessus* isolates had the C28 *erm*(41) variant [30, 31]. In Japan, the geographical distribution and regional differences of the *M. abscessus* group indicated that amongst the *M. abscessus* subsp. abscessus*,* the proportions of C28 sequevar was highly variable, with some regions having 0 % and in other areas as high as 61.5 % [32]. Although presumed to have a positive impact on treatment outcomes, there is limited clinical data on the correlation of the C28 sequevar with outcomes in *M. abscessus* subsp. abscessus*.* Constitutive clarithromycin resistance was not identified in our collection of isolates and parallels our previous observation that constitutive resistance involving *rrl* mutations was rare and seen in 2.2 % isolates (2/90 isolates) [27]. Similarly, in another study, none of the 42 *M. abscessus* isolates exhibited point mutations in the *rrl* gene [33]. Treatment with clarithromycin may select for constitutive mutants over a prolonged treatment duration [34]. Although antibiotic consumption data was not studied here, this may reflect that development of *rrl* mutations are uncommon in the absence of antibiotic selection pressure. There was otherwise no difference in terms of other drug classes’ susceptibility profiles of *M. abscessus* subsp. *abscessus* and *M. abscessus* subsp. massiliense*.* The choice of companion antibiotics therefore would be minimally affected by subspecies identification. *In vitro* data suggest potential for clofazimine, bedaquiline, and eravacycline as new antimicrobial options in treating *M. abscessus* infections [11].

The dominant sequence types seen in our population (* M. abscessus* subsp *. abscessus* ST5; *M. abscessus* subsp *. massiliense* ST4, ST6, ST7, ST10) had been previously identified as clustered STs within the global cystic fibrosis (CF) patient community [4]. The study had involved a large-scale whole genome analysis of a global collection of 1080 clinical *M. abscessus* isolates from 517 CF patients [4]. The predominance of these STs also suggest that these were also locally dominant circulating clones within the community. Closely-related *M. abscessus* was demonstrated, which in conjunction with epidemiological and contact links indicates potential infection control risks present when vulnerable patients come into contact with each other in healthcare settings. To investigate whether the same phenomena occurred in Asian populations and in non-pulmonary infections, we investigated virulence factors and SNPs in our population. Of note, ESX3 appears to be most common in the dominant *M. abscessus* subsp. *massiliense* ST4 and ST6, but otherwise absent from ST7, ST10, and all *M. abscessus* subsp. *abscessus* isolates. There was no predilection to sample type demonstrated. As for other virulence factors investigated, they also appear to be prevalent throughout all subspecies. In addition to ESX3, there may be other virulence factors yet to be characterized, which could contribute to the increased virulence potential seen in clustered *M. abscessus* isolates.

Despite belonging to the same MLST profile and being from the same geographical region, SNP differences in our population were comparatively wider in our patients. Clusters of isolates did not occur in our isolates as seen in CF patients. Investigations of isolates with <20 SNP differences did not demonstrate any clear links between these patients. The sample sources for these isolates were also quite diverse and included pulmonary and non-pulmonary samples, including one blood culture isolate. While healthcare-associated epidemiological links could not be established, a common exposure in the community cannot be excluded. Comparison of genomes with isolates from environmental sources may provide more clarity in the transmission of *M. abscessus* in the community.

We also explored isolates from specific invasive infections which may represent a common infection source. Isolates from patients with bacteraemia and patients with peritoneal-dialysis-associated-peritonitis were reviewed and were demonstrated to be from diverse sequence-types with no clonal infections (Fig. 1). There was only one identified infection of a cardiac device (RGM234; pacemaker infection). Again, these results do not suggest clonal *M. abscessus* infections in our population, and supports these infections have so far been sporadic unrelated events. However, it is important to note that common exposures and risk factors that may still predispose to these infections even when no clear links are established.

We demonstrate that *M. abscessus* cultured from our Asian population were dominated by the same ST profiles seen in global CF populations and that cross-transmission is absent. The infection control risks appear to be largely limited to the vulnerable CF population, indicating infection from environmental sources remains more common than human-to-human transmission. Virulence factors are largely consistent across the subspecies with the exception of clarithromycin susceptibility and ESX-3. Genomic resistance profiling also demonstrates that clarithromycin susceptibility remains the primary distinguishing phenotype between *M. abscessus* subsp. *abscessus* and *M. abscessus* subsp. massiliense*,* and wild-type susceptibility profiles of other antibiotics were similar between the two predominant subspecies.

## Supplementary Data

Supplementary material 1
